# Supplementary figures and images for: Genetic variation associated with PPO-inhibiting herbicide tolerance in sorghum
Source: PLoS One. 2020 Oct 14;15(10):e0233254. doi: 10.1371/journal.pone.0233254 (PMC7556536; doi:10.1371/journal.pone.0233254)

1

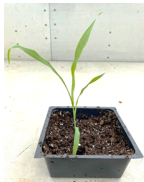

2

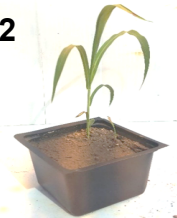

3

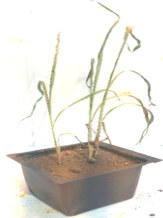

4

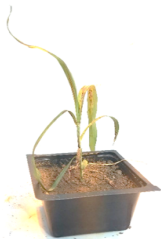

5

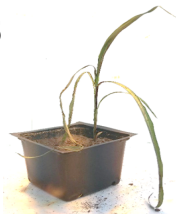

6

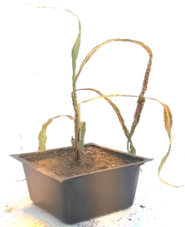

7

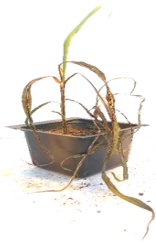

8

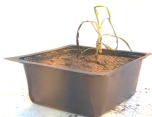

9

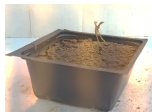

Supplement: S1 Fig — A scale of 1 to 9 was used for visual ratings. Representative photographs of each score are shown. ‘1’ indicates no damage or injury. ‘2’ indicates slight damage (some stunting and chlorosis just visible on lower leaves. ‘3’ indicates slight damage (stunting and chlorotic tissues) obvious on lower leaves, but not persistent. ‘4’ indicates substantial chlorosis on lower and upper leaves that has reached meristematic tissues with stunting, and recovery is possible. ‘5’ indicates strong chlorosis especially on meristematic tissue along with stunting and thinning of stand, and recovery is doubtful. ‘6’ indicates increasing severity, and no recovery possible. ‘7’ indicates increasing severity, and some green tissue still visible. ‘8’ indicates increasing severity with barely any green tissue visible. ‘9’ indicates plant is completely dead with no green tissue. (PDF) [file pone.0233254.s001.pdf]

**A**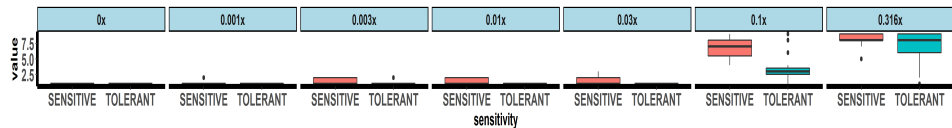**B**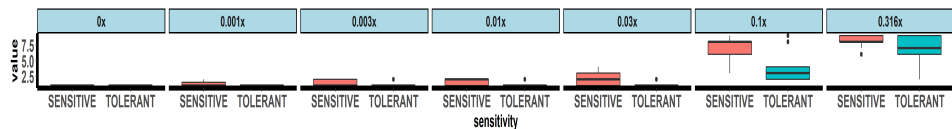**C**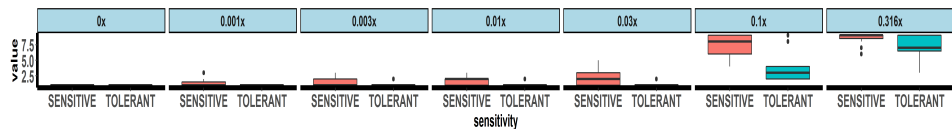**D**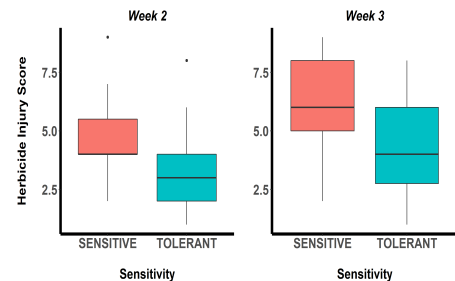**E**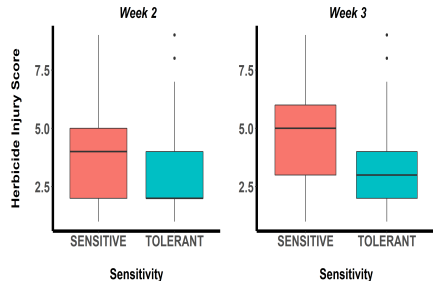

Supplement: S2 Fig — Phenotypic differences between 10 sensitive and tolerant representative sorghum lines selected from the sorghum biomass panel (SBP) for seven herbicide rates at the first week (A), second week (B), and third week (C) after herbicide treatment. Sensitive and tolerant groups were significant at the rate 0.1x fomasefen. Significant phenotypic differences (P<0.0001) were observed in the subset of 10 sorghum lines (D), and 100 sorghum lines (E). (PDF) [file pone.0233254.s002.pdf]
