# Supplementary material for: Genetic variation associated with PPO-inhibiting herbicide tolerance in sorghum
Source: PLoS One. 2020 Oct 14;15(10):e0233254. doi: 10.1371/journal.pone.0233254 (PMC7556536; doi:10.1371/journal.pone.0233254)
Supplement: S1 Table — (DOCX) [file pone.0233254.s003.docx]

**S1 Table.** Primer information for the amplification of PPX1 cDNA.

| **Name** | **Sequence (5'-3')** | **Template**  **strand** | **Start** | **Stop** | **Tm** | **GC%** |
| --- | --- | --- | --- | --- | --- | --- |
| PPO1_1_F | TCGCAGCTCGCAGGGATATG | Plus | 41 | 60 | 62.43 | 60 |
| PPO1_1_R | AGACACCTGAGCAGAAAGGC | Minus | 912 | 893 | 60.61 | 55 |
| PPO1_2_F | TCGGTGCTGAGGTCTTTGAG | Plus | 739 | 758 | 60.18 | 55 |
| PPO1_2_R | GCATTTTTCGGAGGTCACGG | Minus | 1405 | 1386 | 60.25 | 55 |
| PPO1_3_F | ATGGGGAACTCCAGGGTTTT | Plus | 1205 | 1224 | 58.83 | 50 |
| PPO1_3_R | ACTACTCCTGGTCTCACCTCA | Minus | 1733 | 1713 | 59.29 | 52.4 |
| PPO1_4_F | AGTGGAGCGCTGCTTGTTA | Plus | 1676 | 1694 | 59.63 | 52.6 |
| PPO1_4_R | GGCAATCTCAGTTGCACATTCT | Minus | 1976 | 1955 | 59.51 | 45.5 |
