# Supplementary material for: Genetic variation associated with PPO-inhibiting herbicide tolerance in sorghum
Source: PLoS One. 2020 Oct 14;15(10):e0233254. doi: 10.1371/journal.pone.0233254 (PMC7556536; doi:10.1371/journal.pone.0233254)
Supplement: S2 Table — (DOCX) [file pone.0233254.s004.docx]

**S2 Table.** Primer and probe information for the TaqMan assay.

| **Gene** | **Reference Sequence** | **Forward Primer** | **Reverse Primer** | **Probe** |
| --- | --- | --- | --- | --- |
| ***PPXI*** | XM_002455439.2 | GAGGTCTTTGAGCGCCTAAT | CTACCTCCAGCTTCTTCTAACC | AGGCTGCATTTGGGAAGGTGTGGC (Sense) |
| ***PP2A*** | XM_002453490 | AACCCGCAAAACCCCAGACTA | TACAGGTCGGGCTCATGGAAC | ACCCTGATGTTGCGGATGAGAGGAGC (Antisense) |
